# The bacterial capsule is a gatekeeper for mobile DNA

**DOI:** 10.1371/journal.pbio.3001308

**Published:** 2021-07-06

**Authors:** Alfonso Santos-López, Jerónimo Rodríguez-Beltrán, Álvaro San Millán

**Affiliations:** 1 Servicio de Microbiología, Hospital Universitario Ramón y Cajal and Instituto Ramón y Cajal de Investigación Sanitaria, Madrid, Spain; 2 Centro Nacional de Biotecnología–CSIC, Madrid, Spain

## Abstract

The horizontal transfer of mobile DNA is one of the signature moves of bacterial evolution, but the specific rules that govern this transfer remain elusive. In this *PLOS Biology* issue, Haudiquet and colleagues revealed that the interactions between mobile genetic elements and the bacterial capsule shape the horizontal flow of DNA in an important bacterial pathogen.

For billions of years, bacteria have populated almost every corner of Earth, showing an unparalleled ability to adapt to different environments and conditions. One key bacterial adaptation is the capsule, an outer layer of polysaccharides that covers the cells of many different bacterial species. Capsules act as a sort of magic cloak, protecting bacteria from toxic compounds and desiccation and allowing them to adhere to surfaces and to escape the immune system of the host. As a consequence, capsules are important virulence factors, and some capsular types are strongly associated with virulent strains of human pathogens [1].

But how do bacteria acquire ecologically relevant traits such as the capsule? Bacteria are known to engage in horizontal gene transfer (HGT), a process by which bacteria acquire mobile genetic elements (MGEs) carrying specific traits that fuel their evolution. MGEs are specialized vectors that can transfer DNA directly between different bacterial cells. Two of the most relevant types of MGE are bacteriophages (phages), which are viruses that infect bacteria, and conjugative plasmids, which are small circular DNA molecules able to jump from cell to cell through a plasmid-encoded channel. Conjugative plasmids, for example, are the main vehicle for the dissemination of antibiotic resistance genes among bacterial pathogens, which represents an important threat for public health [2].

Being a thick external layer, the bacterial capsule represents an inherent barrier for plasmid and phage transfer. In fact, the capsule is known to prevent phage infection in bacteria by hiding the surface receptors that phages use for invasion [3]. But the capsule/phage interplay is way more complex than that: Phages are able to turn the tide by evolving mechanisms to adhere and invade particular capsular types, producing as a result a strong capsular specificity in phage infections [4].

Phages, plasmids, and capsules are therefore key players for the evolutionary success of pathogens. However, how the interaction between the 2 different types of MGE and the bacterial capsule affects HGT had not been formally investigated till very recently. In a new and elegant study from the Microbial Evolutionary Genomics group (Institut Pasteur, Paris), Haudiquet and colleagues combined sophisticated in silico and in vitro approaches to shed new light on this topic [5]. After comprehensively analyzing ca. 4,000 genomes from the relevant opportunistic pathogen *Klebsiella pneumoniae*, the authors proposed a simple and elegant model to explain the interplay between capsule, phages, and plasmids (Fig 1). First, they showed that, as expected from their high specificity, phages preferentially mediate genetic exchange between bacteria with the same capsule type. Their results also revealed that *K*. *pneumoniae* clones frequently lose their capsule, most likely to avoid phage predation [4]. Next, the authors confirmed that noncapsulated *K*. *pneumoniae* clones are actually more prone to accept conjugative plasmids than capsulated ones, a result that was experimentally confirmed in representative strains of this species. Interestingly, in a previous work by the same group, Rendueles and colleagues described that capsular loci can be encoded in plasmids [6]. These results led the authors to hypothesize that the higher rate of plasmid conjugation in noncapsulated cells could actually promote capsule reacquisition, a hypothesis that they supported with bioinformatic analyses. In summary, the authors described a subtle interplay between phages, plasmids, and *K*. *pneumoniae*, leading to capsule swapping through an intermediate and self-limiting noncapsulated state.

**Fig 1:**
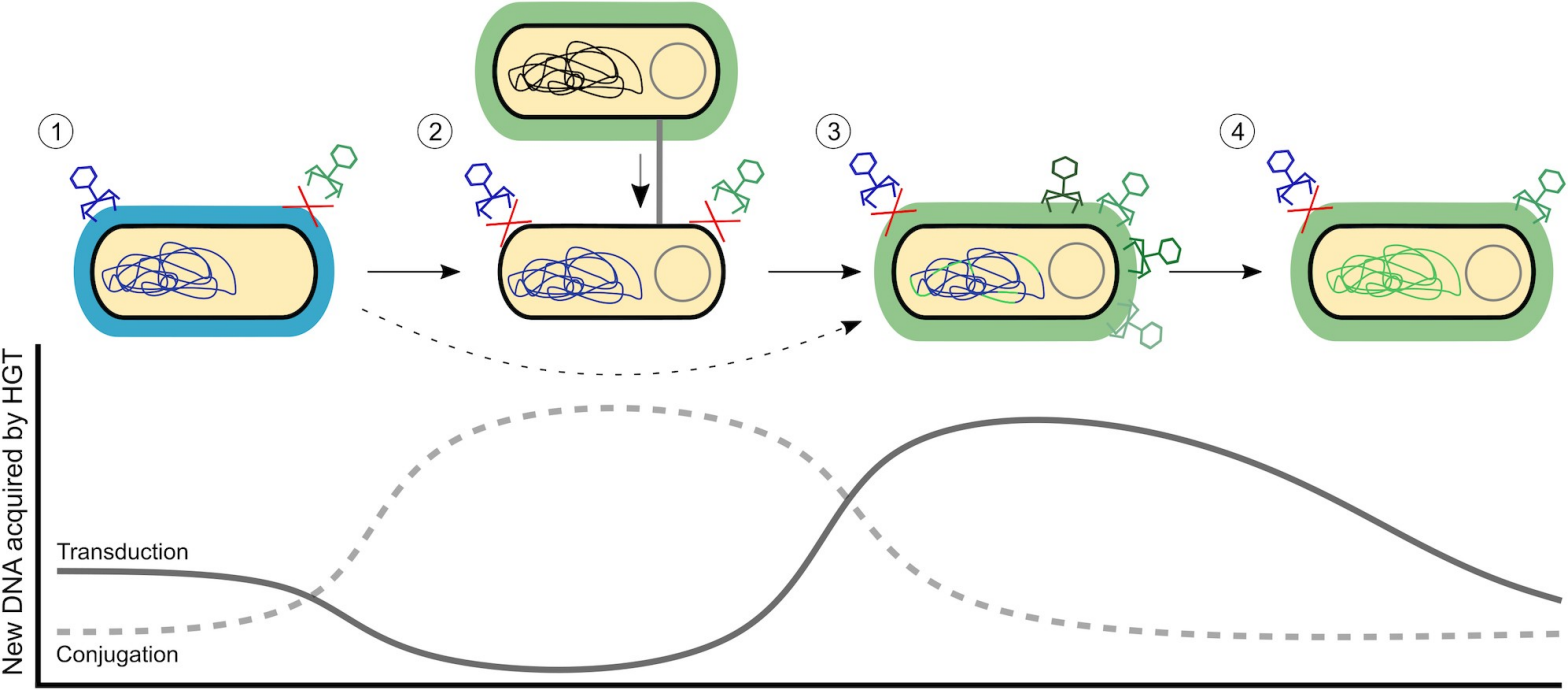
Capsule trading boosts the acquisition of new mobile DNA. The bacterial capsule impacts the horizontal acquisition of novel genetic elements in *K*. *pneumoniae*. (1) Phages are able to transfer DNA between bacteria belonging to the same capsular type (capsule is indicated by blue shading surrounding the bacterium). Capsule inactivation (1→2) leads to resistance to phage infection but increases permissiveness to plasmid reception through conjugation. Conjugative plasmids can promote capsule reacquisition (2→3) or direct swap (1→3; dashed arrow), which can lead to a new capsular type (new capsule is indicated by green shading surrounding the bacterium). Then, new capsule type-specific phages will transfer DNA to the bacterium. The amount of novel DNA transduced by the phages will decay over time (1 and 4), since most DNA exchanges would occur among genetically similar organisms. The lower part of the figure represents a conceptual representation of bacterial access to new horizontally acquired genes through the complete process. Plasmids represent an important source of new genes, while phage-mediated access to novel DNA will be particularly important after changing capsular types, when the bacterium have access to novel phages that bring more diverse DNA (from more phylogenetically distant bacteria). HGT, horizontal gene transfer.

One particularly interesting discovery of the study by Haudiquet and colleagues is the fact that trading capsules—both capsule inactivation and/or capsule swap—is coupled with an increase in the acquisition of MGE (both plasmids and phages) in *K*. *pneumoniae* lineages. As explained before, the authors provide an excellent explanation for the mechanistic basis of this sudden increase in permissiveness to mobile DNA, as well as for its self-limiting nature. It is tempting to speculate that this temporal increase in the flow of new MGE may represent an important opportunity for bacterial evolution (Fig 1). Let us elaborate on this idea; phages readily transfer DNA among bacteria belonging to the same capsular type by generalized, specialized, and lateral transduction (this last type being particularly important for chromosomal hypermobility [7,8]). Apart from those genes required for their lifecycle, phages can encode a reduced number of accessory genes, which may benefit the recipient bacteria. In addition, phages “accidentally” transfer fragments of bacterial chromosome at high frequency, which can be integrated in the chromosomal DNA of the recipient bacteria. Yet, given the high capsular specificity of phages, one may expect that over time, phage-mediated access to novel DNA will decay, since most DNA exchanges would occur among genetically similar individuals. In contrast, capsule trading produces a sudden increase in accessibility to new MGE: conjugative plasmids carrying numerous and novel genes, and phages adapted to a different capsular type, which will bring more diverse DNA. Therefore, as a result of this side effect, capsule trading may be strongly associated with the acquisition of new DNA in *K*. *pneumoniae*, acting as an evolutionary catalyst in this particularly relevant opportunistic pathogen.

The study by Haudiquet and colleagues opens new and exciting research avenues. The most immediate questions involve the particularities of capsule inactivation/swap and their effects on HGT, e.g., How frequently and for how long do *K*. *pneumoniae* lines become noncapsulated? How much more permissive to MGE they become? And how this permissiveness impacts *K*. *pneumoniae* evolution? This study also motivates more general reflections. For example, it highlights the importance of considering the combined effects of different types of MGE when studying HGT. Most of the works on MGE focus on the isolated effects of plasmids, phages, or other MGE. However, it is becoming increasingly evident that different types of MGE operate simultaneously and even coordinately sometimes [9–11], and new studies will help to gain a better understanding how MGE interactions affect bacterial evolution. Finally, this study also emphasizes the impact of the host range of different MGE on their potential effects on bacterial evolution. Phages present a very restricted host range, while plasmids and other conjugative elements, such as integrative and conjugative elements, can spread toward more phylogenetically diverse hosts, ranging from within-species to interkingdom DNA transfer [12]. Future studies will be required to uncover how this different scale of host range may determine not only the ecology but also the genetic cargo of MGE.
